# Beating the odds: Rare prolonged survival of truncus arteriosus: A case report with literature review

**DOI:** 10.1097/MD.0000000000041324

**Published:** 2025-01-31

**Authors:** Obuoma Umejuru Amaewhule, Barbara Edewele Otaigbe, Petronila Nnenna Tabansi, Rosemary Atsosime Uwadiale, Victoria Ezinne Emeruwa, Faithful Miebaka Daniel

**Affiliations:** a Department of Paediatrics, Rivers State University Teaching Hospital, Port Harcourt, Rivers State, Nigeria; b Department of Paediatrics, University of Port Harcourt Teaching Hospital, Port Harcourt, Rivers State, Nigeria; c Community and Clinical Research Division, First On-Call Initiative, Portharcourt, Nigeria; d Community and Clinical Research Division, First On-Call Initiative, Kharkiv, Ukraine; e Department of Public Health, Environment, and Society, London School of Hygiene and Tropical Medicine, London, United Kingdom

**Keywords:** cardiomegaly, case report, echocardiography, hypertension, pulmonary, truncus arteriosus

## Abstract

**Rationale:**

Truncus arteriosus is a cyanotic congenital heart disease in which the great vessels of the heart fail to separate in utero. Consequently, a single truncal vessel arises from the heart to supply the systemic, coronary, and pulmonary circulations. This lesion causes the total mixing of oxygenated and deoxygenated blood, with an early onset of pulmonary vascular disease. Patients rarely survive beyond the first year of life. This case report highlights a rare survival without intervention up to the ninth year of life.

**Patient concerns:**

We present the case of a 9-year-old male child with a history of dark discoloration of the lips and digits, easy fatigability, and fast breathing since birth. The patient is currently small for his age, with a bulging anterior chest wall, displaced apex beat, and cardiac murmur. Oxygen saturation was 57% in room air. A chest radiograph showed peri-hilar lymphadenopathy with prominent pulmonary trunks. Pack cell volume was 62%, and the gene expert test was negative.

**Diagnoses:**

A diagnosis of cyanotic congenital heart disease, truncus arteriosus type III with pulmonary hypertension, was made after diagnostic evaluation with echocardiography.

**Interventions:**

To manage congestive heart failure, the patient was put on diuretics, including furosemide and spironolactone. Parents could not carry out a definitive surgical repair due to financial constraints.

**Outcomes:**

Although the patient was able to survive the past year, the child had developed cardiomegaly, signs of pulmonary congestion, decreased oxygenation, and a compensatory increase in red blood cell volume.

**Lessons:**

Truncus arteriosus is a critical congenital heart defect that has a high potential for morbidity and mortality within the first year of life. It requires immediate intervention and surgical repair in the immediate neonatal period. In rare cases, patients have been able to survive beyond the first year, especially if they also developed pulmonary stenosis. However, this patient had been a survivor for 9 years without another structural anomaly. Given the unique presentation and its rarity, further research is needed into compensatory mechanisms and possible low-cost and accessible alternatives in resource-constrained settings. It also demonstrates that pharmacologic therapy alone is insufficient to prevent mortality.

## 1. Introduction

Truncus arteriosus is a condition that occurs when the 2 great vessels (aorta and pulmonary artery) of the heart fail to completely separate during intrauterine development, leading to an abnormal connection between the aortic and pulmonary circulation.^[1]^ In this condition, there is a mixing of oxygen-rich and oxygen-deficient blood, and consequently, there is an increased pulmonary supply. This eventually increases the work of the heart to perfuse other parts of the body adequately. This structural abnormality is classified as a critical congenital heart defect because it requires intervention and/or surgery within the first year of life, as most (90%) patients will die in infancy.^[2,3]^

Critical CHD screening is recommended within the first 24 hours of life to ensure prompt diagnosis. Pulse oximetry has emerged as a reliable bedside screening tool, which is indispensable for disease recognition in resource-poor settings. This case presentation presents a rare finding of a 9-year-old survivor of truncus arteriosus who has had no medical or surgical interventions. It raises questions about the physiological mechanisms behind prolonged survival and adds to the body of evidence that pharmacological management alone is insufficient to prevent mortality.

## 2. Case presentation

The patient, R.E. was a 9-year-old male who had been experiencing dark discoloration of his lips and fingers, fast breathing, and easy fatigability since birth. However, his parents did not seek any interventions for these symptoms until he presented with the same complaints at 3 years of age. There was no history of smoking, alcohol use, or teratogen exposure in pregnancy; no known medical conditions like gestational diabetes or rubella infection during antenatal screening; and no known family history of congenital heart disease. Parents were of low socioeconomic status. At the initial presentation, a chest radiograph showed signs of cardiomegaly and pulmonary plethora. Unfortunately, R.E. was lost to follow-up, and no interventions were carried out as his parents believed his symptoms would resolve spontaneously.

**Figure 1.**
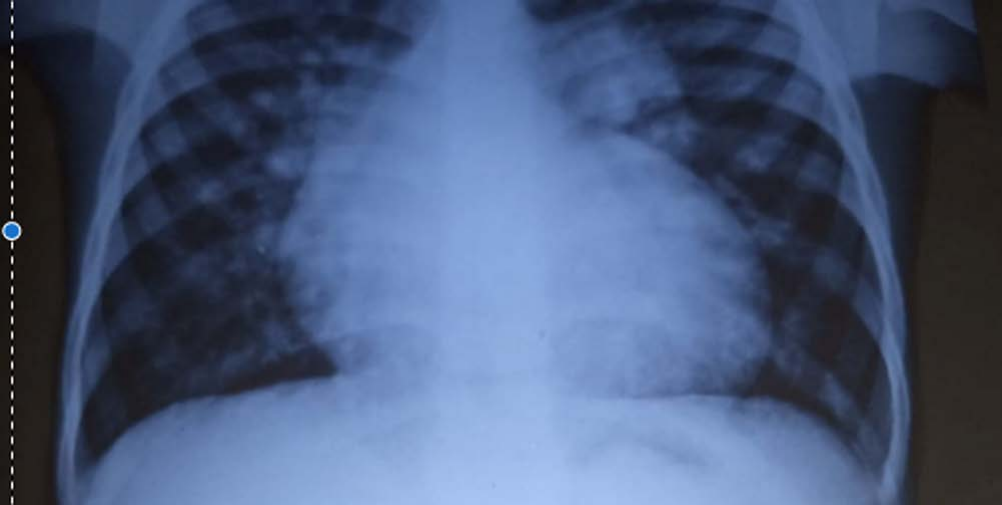
Anterio-posterior chest radiograph showing cardiomegaly, pulmonary plethora, and peri-hilar lymphadenopathy.

**Figure 2.**
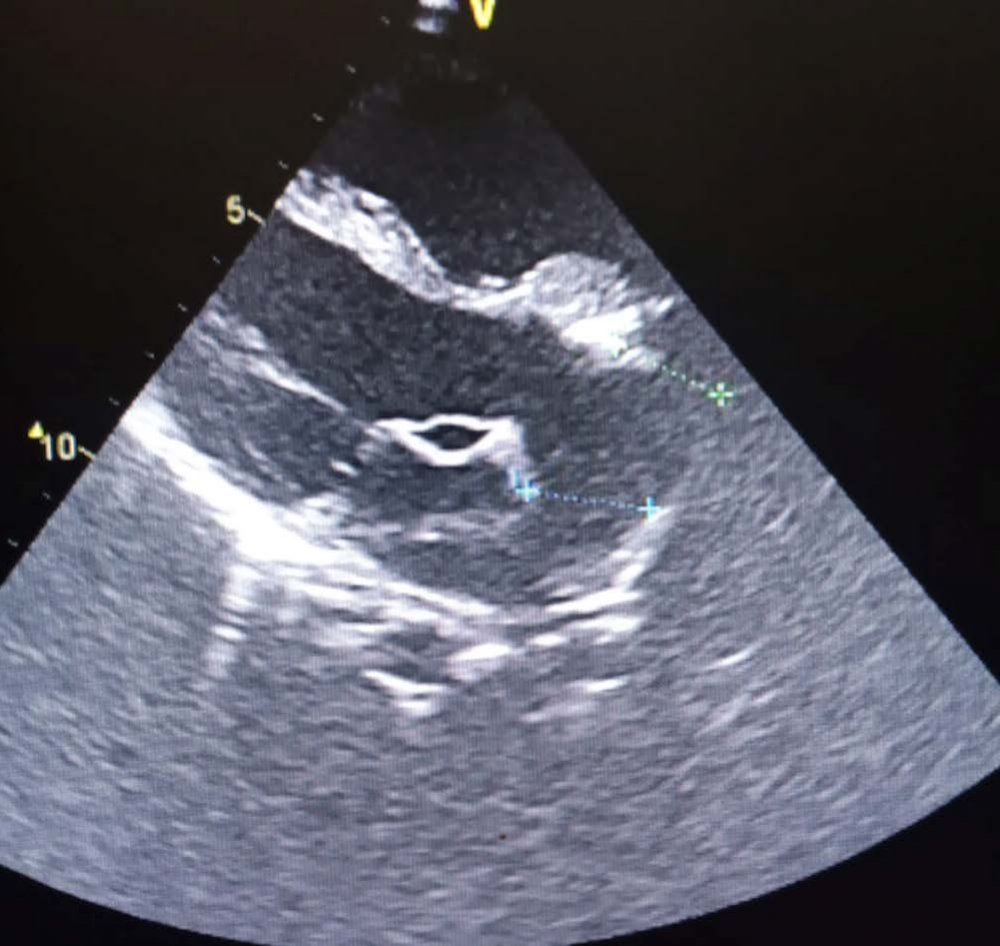
Echocardiographic image showing the aorta arising from the left ventricle and bifurcating into left and right pulmonary trunk.

**Figure 3.**
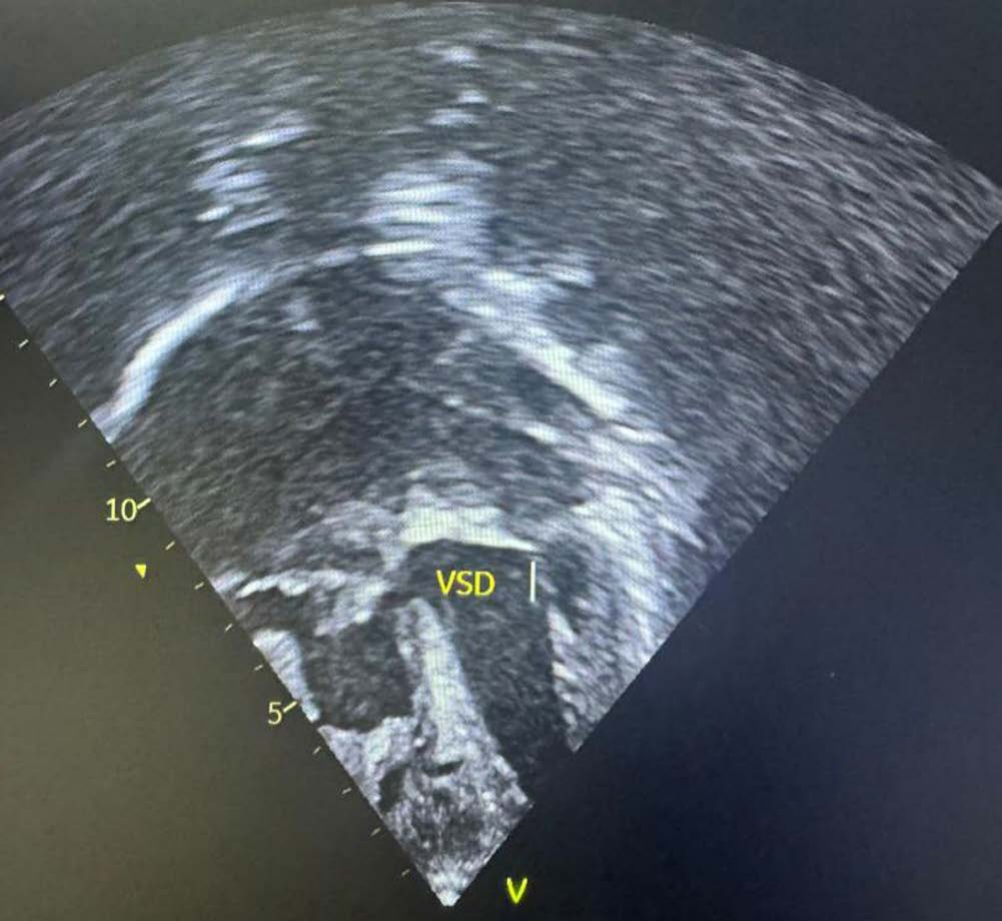
Echocardiographic image showing ventricular septal defect (VSD).

**Figure 4.**
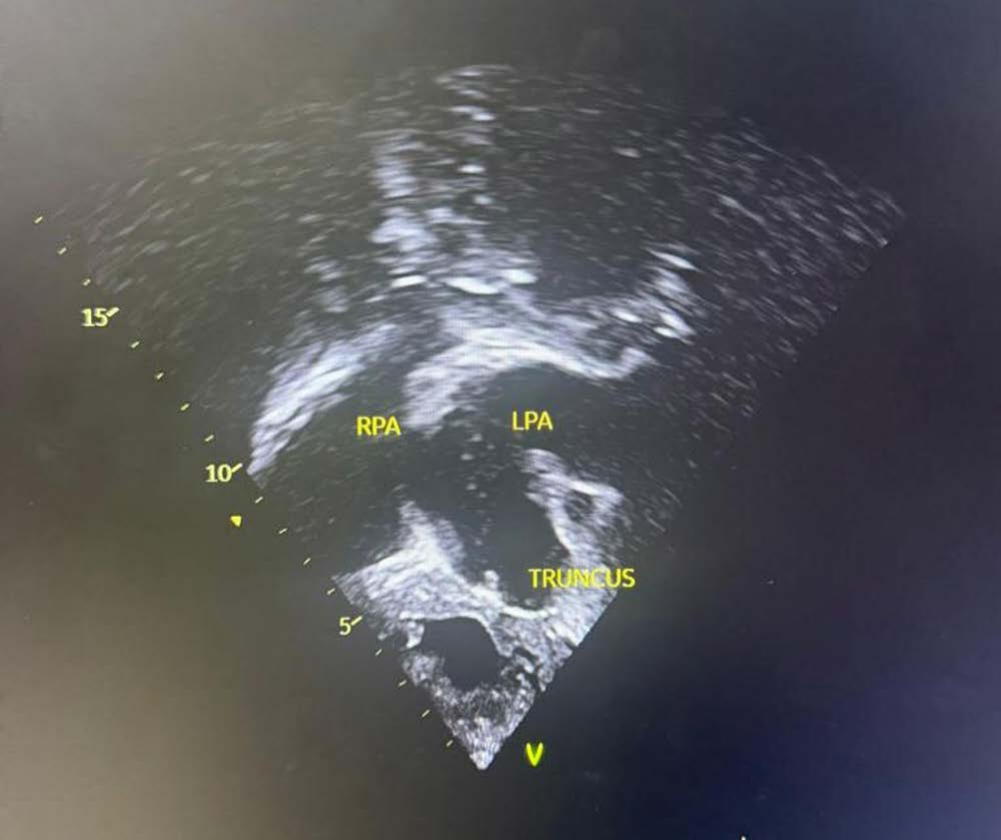
Echocardiographic image showing prominent truncus and right (RPA) and left pulmonary artery (LPA).

**Figure 5.**
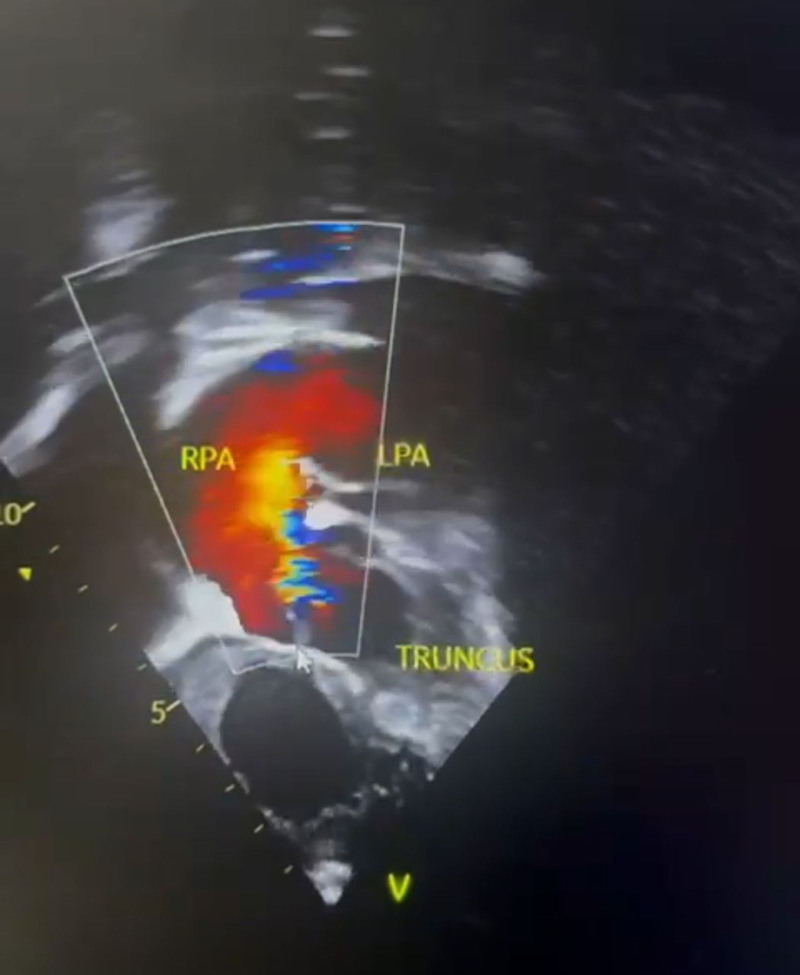
Doppler echocardiographic image showing severity of the ventricular septal defect. LPA = left pulmonary artery, RPA = right pulmonary artery.

Six years later, the patient’s symptoms worsened, and he was brought back to the hospital at the age of 9 years. Upon examination, he was found to be small for his age with a weight of 22 kg (75% of expected) and in respiratory distress, with central and peripheral cyanosis, and oxygen saturation was only 57% in room air. He also had a bulging anterior chest wall, with the apex beat located at the 6th left intercostal space, mid-clavicular line. A grade 3/6 systolic murmur was heard at the tricuspid area, and a loud P2 was audible at the left upper sternal border. A presumptive diagnosis of cyanotic congenital heart disease, likely tetralogy of Fallot, was made. Further investigations revealed a packed cell volume of 62%, and a chest radiograph showed a prominent pulmonary trunk, pulmonary plethora, and cardiomegaly (Fig. 1). However, the gene expert test was negative. Echocardiography revealed that R.E. had a 4-mm restrictive ventricular septal defect (VSD) and a single 20-mm quatricuspid truncal valve, with pulmonary arteries arising laterally. He had eccentric valve regurgitation with pulmonary hypertension (Figs. 2–5). A diagnosis of cyanotic congenital heart disease (truncus arteriosus type III) with pulmonary hypertension was made. The patient had no health insurance, and parents had to cover medical expenses out of pocket. The catastrophic medical expenditure and socioeconomic status of his parents were a barrier to seeking care, posing a significant limitation to diagnostic evaluation and options for surgical therapy. Due to financial constraints, an electrocardiogram, cardiac catheterization, or magnetic resonance imaging of the heart was not done. Pharmacologic therapy was promptly initiated. R.E. was placed on oral diuretics (furosemide 10 mg daily and spironolactone 6.25 mg daily) and phosphodiesterase-5-inhibitor (sildenafil 10 mg twice daily) to manage congestive heart failure and pulmonary hypertension. Parents were counseled that the prognosis was poor without an urgent surgical repair. The importance of a complete surgical repair was emphasized, which may be repeated as the child grows, in order to rebuild the grafted pulmonary artery. Possible complications like heart failure and pulmonary hypertension were discussed with the parents.

Parents were inconsistent with appointments; they were unable to do any follow-up investigations or echocardiography. Regrettably, the patient died from complications of the congenital heart defect, 1 year into follow-up.

## 3. Discussion

Truncus arteriosus is a congenital heart defect first described in 1864 by Buchanan.^[4]^ This critical condition affects approximately 0.8 out of every 10,000 live births and is caused by either complete or partial failure of the development of the aortopulmonary septum. Although it does not show a significant sex predilection, males are more commonly affected.^[5]^ Unfortunately, the true prevalence of truncus arteriosus in low- and middle-income countries like Nigeria is yet to be known due to a lack of prenatal diagnosis, underreporting, scarcity of specialized diagnostic facilities, and poor health-seeking behaviors.^[6]^

The aortopulmonary septum is formed as ingrowths in the wall of the truncus arteriosus, which change position as they progress from distal to proximal.^[1]^ As a result, the septum ends up like a spiral and divides the truncus arteriosus into 2 equal parts: the aorta and pulmonary trunk.^[1]^ However, in persistent truncus arteriosus, the aortopulmonary septum does not form and deoxygenated and oxygenated blood flows into a common trunk.^[1]^

The etiology is multifactorial and may be associated with DiGeorge syndrome, 22q11p2 deletion, maternal diabetes in pregnancy, and teratogens such as retinoic acid.^[7]^ The single trunk has 2 to 6 valves that are stenotic, regurgitant, or both, and a nonrestrictive VSD is always present, enabling the 2 ventricles to empty into a common trunk.^[8]^ Both ventricles work at systemic pressure to eject desaturated blood into this single vessel.^[7]^ Cyanosis is usually mild in truncus arteriosus because of the large volume and blood pressure within the lungs. However, if left untreated, pulmonary vascular resistance (PVR) may develop early, leading to pulmonary hypertension, reduced perfusion, and cyanosis.

Based on the Edwards and Collete classification, truncus arteriosus has 4 types.^[8]^ Type I occurs when the main pulmonary artery originates from the truncus. Type II has the right and left pulmonary arteries originating separately from the posterior surface of the truncus. Type III has the right and left pulmonary arteries originating from the lateral surface of the common trunk. In contrast, type IV has the pulmonary arteries branching off from the descending aorta.^[8]^

The index patient had a type III truncus arteriosus. The clinical features depend on the child’s age and level of PVR. Signs of heart failure and cyanosis are usually absent in the first month of life. With the drop in PVR, signs of heart failure develop in the second month of life. Patients have a hyperdynamic precordium, cardiomegaly, and single loud second heart sounds. Patients typically present in infancy with tachypnea, tachycardia, and failure to thrive.^[9]^

Truncus arteriosus is a condition that can be evaluated through various modalities, which reveal typical features. For instance, a chest radiograph may show an increased cardiothoracic ratio, an aortic arch displaced to the right side in 50% of children, and pulmonary plethora, while an electrocardiogram may show right ventricular hypertrophy. Our patient’s chest radiograph had similar findings in addition to a prominent pulmonary trunk. In addition, echocardiography is diagnostic and can reveal a large truncal artery overriding a ventricular septal defect (Fig. 5). Often, there may be an associated interrupted aortic arch. Doppler studies can also indicate truncal valve regurgitation. Finally, angiography can clearly define the origins of the pulmonary arteries. Overall, these diagnostic modalities work together to identify the features of truncus arteriosus.

It is recommended that patients with truncus arteriosus receive anti-heart failure medication in the first week of life. Surgery should be performed within the first month, and the Rastelli procedure is recommended before 3 months to avoid the development of pulmonary vascular disease. During surgery, the VSD should be closed, and the pulmonary artery separated from the truncus. Continuity should then be established between the right ventricle and the pulmonary artery using a homografted conduit. Although postoperative results are usually excellent, the conduits tend to develop stenosis and regurgitation over time as the child grows and may need to be replaced.

Patients with truncus arteriosus who do not undergo surgery have a poor prognosis, although those with associated pulmonary stenosis may survive longer. Some isolated cases of survival without surgery have been reported, but most of these cases involve pulmonary stenosis, which helps prevent the development of pulmonary vascular disease.^[10]^ For instance, a 35-year-old woman with truncus arteriosus, pulmonary stenosis, and hypoplastic right and left pulmonary arteries was reported, as was a 43-year-old Kenyan male with a stenosed tri-leaflet truncal valve with calcifications.^[11]^ In a Nigerian study by Animasahun et al, 25 children with truncus arteriosus were identified within 9 years, and 7 of them were over 1 year of age. Earlier studies suggest that up to 90% of infants with this condition may die without surgical intervention.^[10]^ The causes of death include metabolic acidosis, cardiac arrest, arrhythmias, myocardial dysfunction, and multiple organ failure.

## 4. Conclusions

Truncus arteriosus is a complex critical cyanotic congenital heart disease in which patients rarely survive beyond the first year of life. This report demonstrates that survival may be longer for some patients. This case raises questions on the possible factors behind prolonged survival for some children as opposed to the experience of the majority of children without interventions. Another key takeaway is that even in the setting of prolonged survival, pharmacologic therapy alone is insufficient to prevent worsening morbidity and mortality. However, measures should be put in place to improve the availability and affordability of surgical repairs in low-resource settings.

## Author contributions

**Conceptualization:** Obuoma Umejuru Amaewhule.

**Investigation:** Obuoma Umejuru Amaewhule, Barbara Edewele Otaigbe, Petronila Nnenna Tabansi.

**Methodology:** Obuoma Umejuru Amaewhule.

**Project administration:** Obuoma Umejuru Amaewhule, Faithful Miebaka Daniel, Victoria Ezinne Emeruwa.

**Supervision:** Obuoma Umejuru Amaewhule, Faithful Miebaka Daniel.

**Writing – original draft:** Obuoma Umejuru Amaewhule, Faithful Miebaka Daniel, Rosemary Atsosime Uwadiale, Victoria Ezinne Emeruwa.

**Data curation:** Faithful Miebaka Daniel, Victoria Ezinne Emeruwa.

**Formal analysis:** Faithful Miebaka Daniel.

**Resources:** Faithful Miebaka Daniel.

**Validation:** Faithful Miebaka Daniel, Victoria Ezinne Emeruwa.

**Visualization:** Faithful Miebaka Daniel.

**Writing – review & editing:** Faithful Miebaka Daniel, Victoria Ezinne Emeruwa.
